# Polymerogenic neuroserpin causes mitochondrial alterations and activates NFκB but not the UPR in a neuronal model of neurodegeneration FENIB

**DOI:** 10.1007/s00018-022-04463-3

**Published:** 2022-07-21

**Authors:** E. D’Acunto, L. Gianfrancesco, I. Serangeli, M. D’Orsi, V. Sabato, N. A. Guadagno, G. Bhosale, S. Caristi, A. V. Failla, A. De Jaco, E. Cacci, M. R. Duchen, G. Lupo, G. Galliciotti, E. Miranda

**Affiliations:** 1 Department of Biology and Biotechnologies ‘Charles Darwin’, Sapienza University of Rome, P.le Aldo Moro 5, 00185 Rome, Italy; 2 Department of Cell and Developmental Biology, University College London, London, UK; 3 University Medical Center Hamburg-Eppendorf, Hamburg, Germany; 4 Pasteur Institute-Cenci Bolognetti Foundation, Sapienza University of Rome, Rome, Italy

**Keywords:** Serpin polymers, Protein conformational disease, Neurodegeneration, ER overload, MERCs, Oxidative stress, Neural progenitor cells, Neurons

## Abstract

The neurodegenerative condition FENIB (familiar encephalopathy with neuroserpin inclusion bodies) is caused by heterozygous expression of polymerogenic mutant neuroserpin (NS), with polymer deposition within the endoplasmic reticulum (ER) of neurons. We generated transgenic neural progenitor cells (NPCs) from mouse fetal cerebral cortex stably expressing either the control protein GFP or human wild type, polymerogenic G392E or truncated (delta) NS. This cellular model makes it possible to study the toxicity of polymerogenic NS in the appropriated cell type by in vitro differentiation to neurons. Our previous work showed that expression of G392E NS in differentiated NPCs induced an adaptive response through the upregulation of several genes involved in the defence against oxidative stress, and that pharmacological reduction of the antioxidant defences by drug treatments rendered G392E NS neurons more susceptible to apoptosis than control neurons. In this study, we assessed mitochondrial distribution and found a higher percentage of perinuclear localisation in G392E NS neurons, particularly in those containing polymers, a phenotype that was enhanced by glutathione chelation and rescued by antioxidant molecules. Mitochondrial membrane potential and contact sites between mitochondria and the ER were reduced in neurons expressing the G392E mutation. These alterations were associated with a pattern of ER stress that involved the ER overload response but not the unfolded protein response. Our results suggest that intracellular accumulation of NS polymers affects the interaction between the ER and mitochondria, causing mitochondrial alterations that contribute to the neuronal degeneration seen in FENIB patients.

**Supplementary Information:**

The online version contains supplementary material available at 10.1007/s00018-022-04463-3.

## Introduction

Neurodegeneration is often characterised by the aggregation of misfolded proteins within neurons, a fact that led to the grouping of these pathologies as conformational dementias [1]. This group includes the autosomal dominant condition known as familial encephalopathy with neuroserpin inclusion bodies (FENIB, [2]). Although its name implies family transmission, several of the known mutations in neuroserpin (NS) that cause FENIB, and particularly those that cause severe disease, occur as sporadic cases [3–6]. These mutations drive polymer formation by mutant NS within the endoplasmic reticulum (ER) of neurons of the central and peripheral nervous system, giving rise to inclusion bodies and causing neurodegeneration that underlies a range of symptoms, including cognitive decline, dementia, myoclonus and epilepsy, ending with the death of the patient [2–6]. The mutations that introduce higher instability in the structure of NS result in earlier age of onset and increased severity of the clinical symptoms (with severity order S49P < S52R < H338R < G392E), in a remarkable correlation between genotype and phenotype [3] that was confirmed by later work in cell culture and fly models of FENIB [7].

NS is a serpin secreted from neurons across the developing and adult nervous system, with roles in neuronal differentiation, neurite extension and synapse formation and plasticity, but also in immune cell function [8, 9]. Fly and mouse models of FENIB have shown the formation of neuronal inclusions made of NS polymers and phenotypes reminiscent of FENIB in human patients, supporting the toxicity of polymerogenic neuroserpin expression even in the presence of the wild type, endogenous NS gene [7, 10, 11]. In contrast, several cell culture models based on transformed cell lines have failed to show evident signs of cell toxicity upon polymerogenic NS expression [7, 12–16]. This led us to create a neuronal model of FENIB based on in vitro differentiation of mouse neural progenitor cells (NPCs), that we used to investigate the effects of polymerogenic NS in neurons. We found that expression of G392E NS, which is associated with severe FENIB [3], caused the upregulation of genes involved in the defence against oxidative stress. Moreover, G392E NS neurons showed increased apoptosis in response to oxidative insults [17].

A wealth of the research supports an important role for oxidative stress and the mitochondrial network, the major organelle involved in oxidative stress, in the pathological mechanisms of neurodegenerative diseases, often as a direct consequence of mutations on proteins involved in mitochondrial dynamics [18, 19]. Furthermore, it is becoming increasingly clear that all intracellular organelles are interconnected in a dynamic network that sustains cellular homeostasis and undergoes constant changes under different physiological and pathological conditions, in what has been called the cell *communicome* [20]. The first connection that was described between organelles involves the ER and mitochondria, whose membranes become close, without joining, at areas first known as ER mitochondria-associated membranes (MAMs, [21]) and functionally renamed mitochondria-ER contact sites (MERCs). MERCs are of different types depending on the proteins recruited to the contact site and thus participate in different activities where both the ER and mitochondria take part, like lipid exchange, Ca^2+^ transfer and redox signalling, but also in complex processes including mitochondrial dynamics and mitophagy [22].

In non-neuronal cell lines, the accumulation of NS polymers within the ER causes a type of ER stress that is different from the classical UPR (unfolded protein response) and shows no activation of the conventional signalling branches: PERK (protein kinase R-like ER kinase), IRE1α (inositol-requiring enzyme 1 α) and ATF6 (activating transcription factor 6) [13]. This response is specific to polymerogenic NS, since delta NS, a truncated version of the protein that cannot complete a proper folding, behaves as a *bonafide* misfolded protein, gets efficiently degraded by ERAD (ER associated degradation) and activates the UPR when its degradation is prevented by pharmacological inhibition of the proteasome [13]. Instead, ER accumulation of NS polymers triggered a Ca^2+^-dependent activation of NFκB that was similar to the previously described consequence of overloading the ER with viral proteins, the so-called ER overload response (EOR, [23]). Activation of the EOR has also been found upon accumulation of polymers of α-1 antitrypsin (A1AT) in the ER, again in the absence of UPR activation [24], giving support to the hypothesis that accumulation of ordered polymers of mutant serpins within the ER creates a characteristic pathological condition that differs from many other disorders involving protein misfolding and aggregation. The exact mechanism that leads from ER polymer accumulation to the Ca^2+^-mediated activation of NFκB and to neuronal toxicity is still unknown.

Here we build on our previous studies in a neuronal system in vitro to elucidate the cellular responses to NS polymers formed by the G392E NS variant, using GFP (overexpression of a heterologous protein), WT NS and delta NS (a NS mutant that does not polymerise) as controls. After showing that polymer accumulation causes oxidative stress [17], we now find that neurons overexpressing G392E NS present mitochondrial alterations, in particular a relocalisation to the perinuclear zone and a decrease in the inner mitochondrial membrane potential. We also show that the perinuclear localisation of mitochondria is enhanced by depletion of glutathione and rescued by antioxidant treatments, and that G392E NS neurons have a decrease in contacts between mitochondria and the ER. Finally, we confirm in our neuronal model that NS polymers do not activate the UPR but trigger the EOR.

## Materials and methods

### Reagents and antibodies

Unless otherwise stated, buffers, reagents, culture media and serum for cell cultures were purchased from Merck|Sigma-Aldrich. Rabbit polyclonal anti-NS antibody and mouse monoclonal anti-NS antibodies were made in-house as reported before [7]. Mouse anti-ß actin (MAB1501), mouse anti-nestin (MAB5326) and mouse anti-VDAC1 (MABN504) antibodies were from Millipore; mouse anti-ßIII tubulin (G712A) was from Promega; rabbit anti-TOM20 (sc-17764), mouse anti-IP3R-I (SC-271197) and mouse anti-sigma-1 receptor (SC-137075) antibodies were from Santa Cruz Biotechnology; mouse anti-KDEL antibody (#ADI-SPA-827) was from Enzo Life Science; rabbit anti-phosphorylated-eIF2α (Ser51) (#119A11) and mouse anti-total eIF2α (#L57A5) antibodies were from Cell Signalling. Goat anti-rabbit-HRP (horseradish peroxidase) (A0545), goat anti-mouse-HRP (A9044) and goat anti-mouse Aberrior STAR® 635P (07679) antibodies were from Merck|Sigma-Aldrich. Goat anti-mouse IgG-Alexa Fluor® 488 (150113) and -Alexa Fluor® 594 (15116), and goat anti-rabbit IgG-Alexa Fluor® 594 (150080) and Alexa Fluor® 488 (150077) were from Abcam.

### Generation of neural progenitor cell lines with stable expression of neuroserpin

This work was carried out by in vitro culture of available liquid nitrogen stocks of mouse NPCs that were previously derived from the developing cerebral cortex of embryonic day 13.5 (E13.5) mouse embryos. The original derivation of mouse cortical NPCs was performed in accordance with EU and Italian regulations and with ethical approval by the Ethical Committee for Animal Research of the Italian Ministry of Health, as described [25]. No additional animals were employed for the experiments reported in the present study. The cell lines used in this study were newly generated following the protocol described before [17]. Briefly, early passage NPCs obtained from embryonic cortex of E13.5 mouse cerebral cortex were transfected with pTP6-NS plasmids containing a puromycin resistance gene and the gene sequence for WT, G392E or delta NS. The last is a truncated version of NS lacking the last 134 amino acids and used as a misfolding protein control [13]. NPCs were transfected with Amaxa Nucleofector using the Neural Stem Cell Nucleofector kit (Lonza, VPG-1004) according to the manufacturer’s instructions. Two days after transfection, NPCs with stable integration of the pTP6-NS transgenes were selected and expanded by adding puromycin (P8833) at 1 µg/ml to the culture medium. A control NPC line expressing cytosolic GFP was previously described [17] and was used here as a negative control for non-specific effects due to our overexpression approach.

### Neural progenitor cell cultures and treatments

Cells were cultured in proliferative conditions in T25 flasks coated with 10 µg/ml poly-ornithine (P-3566) and 5 µg/ml laminin (354232), in basal medium [DMEM/F12 (Invitrogen, 32500035), penicillin/streptomycin (P4333, 1/100), 200 mM Glutamax (Invitrogen, 35050038), 10 mM Hepes (H0887), 0.892 M NaHCO_3_ (S8761), 0.033 M D-glucose (G8769)] supplemented with 1 µg/ml puromycin, 20 ng/ml human recombinant epidermal growth factor (R&D, hEGF, 236-EG-01 M), 10 ng/ml human recombinant basic fibroblast growth factor (R&D, bFGF, 4114-TC-01 M), 2% v/v B-27 (Life Technologies, 17502048) to make complete medium. Upon confluence, cells were dissociated with Accutase (25-058-CI) and re-seeded on fresh flasks at a density of 10,000 to 20,000 cells/cm^2^. Cells were kept at 37 °C in a 5% CO_2_ atmosphere. For neuronal differentiation, NPCs were plated at a density of 24,000 cells/cm^2^ in complete medium; 24 h later this was replaced with neuronal differentiation medium [basal medium containing 10 ng/ml bFGF, 1% v/v N2, 2% v/v B-27, 0.5 µM DAPT (Tocris Bioscience, 2634) and 40 ng/ml human brain derived neurotrophic factor (hBDNF, Peprotech, 450-02-50UG), which was kept for 6 days with a change of medium at day 3. After 6 days under these conditions, NPCs differentiated into a mixed culture of neurons and partially differentiated cells (as reported in [26] and shown in the “Results” section).

For pharmacological treatments, NPCs were cultured in differentiation medium for 6 days in 24-well plates pre-treated with poly-ornithine and laminin as described above. When treating with hydrogen peroxide (H_2_O_2_) (H1009) or diethyl maleate (DEM) (W505005), at day 5 cells were treated with 100 µM H_2_O_2_ for 30 min or 50 µM DEM at 37 °C for 1 h. After the incubation, culture medium was replaced with fresh medium and cells cultured for the last 24 h at 37 °C. When treating with α-tocopherol (T3251) or melatonin (M5250), from day 4 cells were incubated in the presence or absence of 10 µM tocopherol or melatonin for the last 48 h of differentiation. For luciferase and real time reverse transcription polymerase chain reaction (RT-PCR) assays, NPCs were cultured in differentiation medium for 3 days in 6-well plates or for 6 days in T25 flasks, respectively, both pre-treated with poly-ornithine and laminin as described above. At day 4, cells were treated with 2.3 µg/ml of tunicamycin (T7765) or 10 ng/ml of IL-1ß (R&D, 501-RL) at 37 °C for 6 h as positive controls for UPR and NFκB activation respectively.

### RNA extraction, reverse transcription and polymerase chain reaction

Proliferative and differentiated NPCs cultured in T25 flasks were lysed in 400 µl/flask of RLT buffer and total RNA was extracted following the kit’s instructions (Qiagen, RNeasy Mini Kit, 74,104). To increase the recovery of total RNA from differentiated cells, a carrier RNA was added to the RLT buffer and 20 µl of RNase-free water were used for RNA elution. RNA was quantified with a Thermo Scientific NanoDrop 2000 spectrophotometer. For real-time RT-PCR, 1 µg of each RNA sample was reverse-transcribed using the Qiagen QuantiTect reverse transcription kit (235311) and amplified by real-time RT-PCR on a Rotor-Gene Q (Qiagen), using a Qiagen QuantiFast SYBR Green PCR kit (204057). Primers sequences for *Dcx* were previously described [27]. The following primers were used to detect unspliced *XBP1*:

Forward primer: 5′-CAGCACTCAGACTATGTGCACCTC-3′.

Reverse primer: 5′-GCCCAAAAGGATATCAGACTCAGAA-3′.

Relative gene expression levels were determined with the built-in comparative quantitation method using *Rpl19* as normalizer [25].

### Cell sample preparation and protein quantification

Cell lysates from differentiated cells were obtained by adding 80 µl of complete lysis buffer (150 mM NaCl, 50 mM TRIS–HCl pH 7.5, plus 1% v/v Nonidet P-40 (492016) and 1X protease inhibitors cocktail) directly into the T25 flask, using a cell scraper (Corning, 353086) for cell detachment and lysis, followed by recovering of the lysis volume and incubation for 20 min on ice. For anti-phosphorylated and anti-total eIF2α immunoblot, phosphatase inhibitors (P2714-1BLT, 50 µM NaF, 1 µM Na3VO4, 1 µM PMSF) were added to the same lysis buffer. The lysate was cleared by spinning at 17,000*g* in a benchtop centrifuge at 4 °C for 15 min. Total protein concentration of cell lysates was measured by Bradford assay (Bio-Rad, 5000006) following the manufacturer’s instructions, using bovine serum albumin (BSA, A4503, stock 1 mg/ml) for the standard curve. The absorbance was measured in a plate reader (Thermo Fisher Multiskan FC) at 595 nm. For culture medium supernatant analysis, samples were centrifuged at 500*g* for 5 min at RT to remove dead cells and cell debris.

### Denaturing and non-denaturing polyacrylamide gel electrophoresis and western blot analysis

Samples from differentiated cells had very low protein concentrations, so the maximum volume (30 µl) was loaded per lane. Cell lysates were mixed with non-denaturing loading buffer or loading buffer containing ß-mercaptoethanol (444203) and sodium dodecyl sulphate (SDS, L4390) plus heating at 95 °C for 10 min for denaturing gel electrophoresis, and analysed by polyacrylamide gel electrophoresis [PAGE, w/v acrylamide (A3699) of 10% for denaturing and 7.5% for non-denaturing PAGE] and western blot, following protocols published before [12, 28], except no methanol was added to the transfer buffer for denaturing analysis. Membranes were blocked with 5% (w/v) non-fat dried milk powder in PBT (2.68 mM KCl, 1.47 mM KH2PO4, 0.137 M NaCl, 10.16 mM Na2HPO4, 0.1% v/v Tween20). Primary antibodies were diluted in PBS with 3% BSA and 0.1% sodium azide at the following dilutions: polyclonal rabbit anti-NS antibody (whole serum) diluted 1:10,000; mouse anti-IP3R-I 1:200; mouse anti-Sigma receptor 1:200; mouse anti-VDAC1 1:200; mouse anti-KDEL 1:1,000; rabbit anti-P-eIF2α 1:1,000; mouse anti-total eIF2α 1,1000; and rabbit polyclonal anti-ß actin used as a loading control 1:1,000. Primary antibodies were detected with anti-rabbit IgG- or anti-mouse IgG-HRP secondary antibodies diluted at 1:40,000 in PBT plus 5% (w/v) non-fat dried milk powder, and the signal was developed with ECL substrates [Euroclone LiteAblot Plus Enhanced Chemiluminescent Substrate (EMP01005) or LiteAblot Turbo Extra-sensitive Chemiluminescent Substrate (EMP012001)] using a ChemiDoc instrument (BioRad). Densitometry was performed on at least 3 independent experiments using ImageJ software (NIH).

### Sandwich ELISA

Quantification of polymeric NS was carried out by using a monoclonal antibody with high affinity for NS polymers (7C6) in sandwich ELISA as described before [7, 28], with the conditions described here in brief. 96-Well plates (Corning, CLS9018BC) were coated overnight at room temperature with 50 µl of antigen-purified rabbit polyclonal anti-NS antibody at 4 µg/µl in PBS. Wells were washed 3 times with washing buffer (0.9% w/v NaCl, 0.05% v/v Tween 20) and blocked for 2 h with 300 µl/well of blocking buffer (PBS, 0.25% w/v BSA, 0.05% v/v Tween 20, 0.01% w/v sodium azide). Standards (recombinant purified mutant NS polymerised by heating in vitro) and cellular samples diluted in blocking buffer were incubated for 1 h at 37 °C, washed, incubated with 7C6 antibody (1 µg/ml in blocking buffer) for 1 h at 37 °C, washed and incubated with rabbit anti-mouse HRP antibody (1:20,000 in blocking buffer without sodium azide) for 80 min at RT. After developing for 10 min at RT with TMB substrate solution (T0440) and stopping the reaction with 1 M H_2_SO_4_, the product of HRP activity was measured in a Thermo Fisher Multiskan FC plate reader at 450 nm.

### Fluorescence staining and microscopy

NPCs were grown in 24-well plates on coverslips pre-treated with poly-ornithine and laminin and differentiated for 6 days as described above. For mitochondrial distribution analysis, cells were incubated with Mitotracker Red CMX Ros ® (Life Technologies, M7512) diluted at 100 nM in culture medium for 10 min at 37 °C, washed in pre-warmed PBS, fixed in 4% paraformaldehyde (PFA) at RT for 15 min, permeabilised with 0,1% Triton X-100 in PBS for 10 min, washed and counterstained with 6-diamidino-2 phenylindole (DAPI, D9542) for 10 min and finally mounted with Fluorosave (Calbiochem, 345789-20) containing 2% 1,4-diazabicyclo [2.2.2] octane (DABCO, D2,780-2). Mitochondrial distribution was analysed with an Apotome fluorescence imaging system (Zeiss) at 63× magnification. At least one hundred fifty cells were evaluated for each condition and mitochondrial distribution was classified into three categories as described in the Results section.

For immunofluorescence staining, differentiated NPCs were rapidly washed in pre-warmed PBS, immediately fixed in 4% PFA for 15 min, washed and permeabilised as above, incubated for 1 h with blocking solution (0.1% Triton, 5% normal goat serum, 0.1% sodium azide in PBS) and next with the primary antibodies: rabbit anti-TOM20 (1:60), mouse anti-ßIII tubulin (1:400), mouse anti-nestin (1:200) or mouse anti-NS polymers 7C6 (10 µg/ml), overnight at 4 °C. After washing, cells were incubated with the appropriate secondary antibodies (anti-rabbit-Alexa 594, anti-mouse Alexa 488 and anti-mouse Aberrior STAR 635P, 1:500) for 60 min in blocking solution, washed and mounted in Fluorosave plus 2% DABCO. Stained samples were analysed with an Apotome fluorescence imaging system (Zeiss) and with a STED microscope at 63× magnification. Imaris Microscopy Software and ImageJ software (NIH) were used for quantification analysis.

### Evaluation of inner mitochondrial membrane potential with JC-1 cationic dye

NPCs were grown on coverslips as above. After 6 days of differentiation, cells were stained with the JC-1 Mitochondrial Membrane Potential Detection Assay Kit (Biotum, 30001-T) at a 1:100 dilution for 15 min. After a wash in PBS, cells were mounted on slides adding a small drop of PBS to facilitate adhesion and living cells were analysed by confocal microscopy (Zeiss 780) with 63× magnification. JC-1 aggregates (red fluorescence) and monomers (green fluorescence) were imaged simultaneously with a dual band-pass filter for fluorescein (Ex/Em 490/520 nm) and rhodamine (Ex/Em 540/570 nm). The confocal images were analysed for fluorescence quantification as the ratio of aggregate/monomer using the image analysis software Image J, analysing at least 100 neurons for each independent experiment.

### Seahorse assay

Oxygen consumption rates (OCR) were measured with the Seahorse XFe96 Analyser (Agilent) using the Seahorse XFe96 FluxPak (Agilent, 102416-100). NPCs were plated on Seahorse XF96 cell culture microplates (pre-coated as above) at 8000 cells/well on day 0, then differentiated for 6 days as described above. The XFe sensor cartridge was hydrated overnight using the Seahorse XF Calibrant Solution at 37 °C in a 0% CO_2_ incubator the day before the experiment. On the assay day, cells were washed twice and incubated with 175 µL of recording medium, prepared using Seahorse XF base medium (102353-100, Agilent), 1 g/L glucose, 1 mM sodium pyruvate and 2 mM glutamate, for 1 h in a 0% CO_2_ incubator. In the meantime, the hydrated XFe sensor cartridge was loaded with 25 µL of 1 µM oligomycin (O4876) in port A, 25 µl of 1 µM of carbonyl cyanide-*p*-trifluoro methoxyphenyl hydrazone (FCCP, C2920) in port B and port C and 25 µl of 2.5 µM antimycin A (A8674) in port D. The loaded cartridge and the utility plate were placed in the Seahorse XFe96 Analyzer for calibration and, after the replacement of the utility plate with the one containing the cells, The Seahorse XF Mito Stress assay was performed following this protocol: (1) equilibrate: 18 min; (2) OCR reading: 3 cycles of 3 min Mix, 0 min Wait and 3 min Measure; (3) 1 µM oligomycin injection; (4) OCR reading as in step 2; (5) 1 µM FCCP injection; (6) OCR reading as in step 2; (7) 1 µM FCCP injection; (8) OCR reading as in step 2; (9) 2.5 µM antimycin A injection; (10) OCR reading as in step 2. After the assay, cells were stained with 2.5 µM Hoechst (Thermo-Fisher Scientific) for 10 min and counted using the ImageXpress Micro XLS Widefield High-Content Analysis System.

### Luciferase assays

NPCs were transfected in 6-well plates with Amaxa Nucleofector using the Neural Stem Cell Nucleofector kit according to the manufacturer’s instructions, with plasmids containing the firefly luciferase reporter gene p(5X)ATF6-luc for UPR activation or the firefly luciferase reporter gene pELAM1-luc for NFκB activation; the Renilla luciferase gene pRL-TK was used as a transfection control as reported before [13]. After 24 h, the proliferative medium was replaced with neuronal differentiation medium, and luciferase activity was measured 72 h later using the Dual Luciferase Reporter Kit Assay (Promega, E1910). Cells were washed with PBS and lysed with 50 µl of Passive Lysis Buffer 1X for 15 min at RT under gentle stirring. After scraping, the cell lysates were collected and centrifuged at 17,000*g* in a benchtop centrifuge for 30 s to remove cell debris. The supernatants (20 µl) were loaded in duplicate wells in a 96-well luminescence plate and the assay performed in a Glomax luminometer (Promega), using the injectors to add 70 µl of the LAR (Luciferase Assay Reagent) and 70 µl of the Stop & Glo reagents to each well. Firefly luciferase signal was normalized to control Renilla luciferase signal for each well.

### Figure preparation and statistical analysis

All experiments were performed at least three times as independent replicates. The results of all experiments are expressed as the mean ± standard deviation (SD). Graphing and statistical analysis of the data were conducted using the t-student test or ANOVA by using Prism5 (Graph-Pad 5 Software Inc.) or Microsoft Excel. Differences were considered significant with *p* values of *p* ≤ 0.05 (*), *p* ≤ 0.01 (**) or *p* ≤ 0.001 (***)and only significant differences are reported in the figures. The figures were prepared using the free software Inkscape.

## Results

### Generation and characterisation of transgenic NPCs overexpressing WT and mutant neuroserpin

**Fig. 1:**
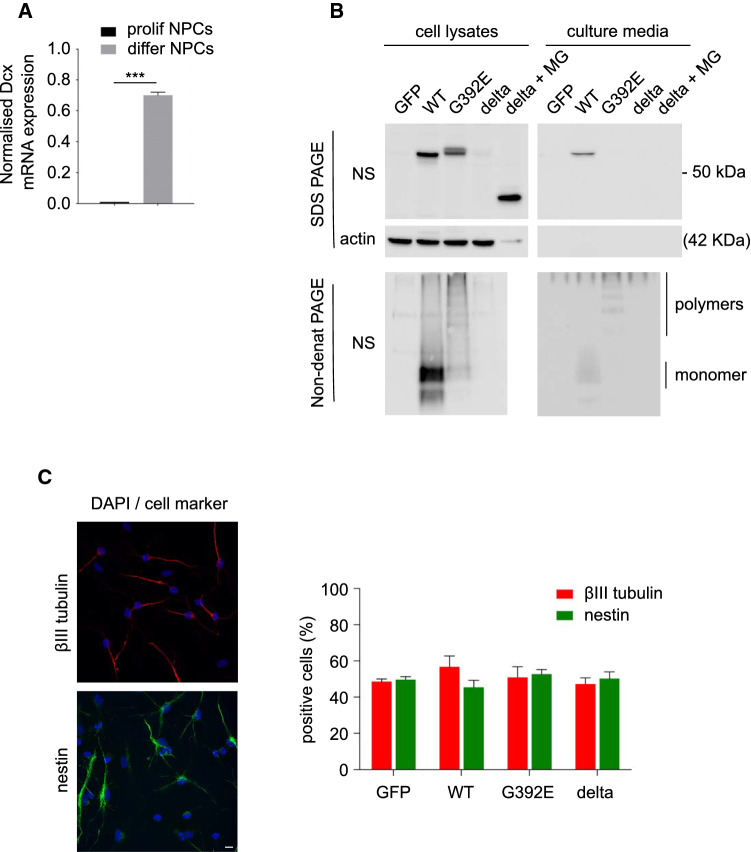
Neural progenitor cells differentiate to neurons under chemically defined conditions. **A** Expression of the neuroblast differentiation gene *Dcx* in NPCs cultured under proliferating and differentiating conditions, normalised to expression in explants of E13.5 mouse telencephalon. The data are mean ± SEM of 3 independent experiments, *t* test: ***p ≤ 0.001. **B** Cell lysates and culture media of stably transfected NPCs expressing GFP or WT, G392E or delta NS, were collected after 6 days of differentiation. Delta NS cells were treated or not with the reversible proteasomal inhibitor MG132 (2 µM for 12 h) to demonstrate expression, since this variant is efficiently degraded by ERAD [13]. Samples were resolved by 10% w/v acrylamide SDS-PAGE (top panels, including culture medium for delta NS ± MG132, which did not show NS reactivity since this variant is not secreted [13]) or 7.5% w/v acrylamide non-denaturing PAGE (bottom panels, delta NS lysate and culture medium were included only without MG132, since this variant does not polymerise [13]) and analysed by western blot with an anti-NS polyclonal antibody. The same SDS-PAGE membrane was probed for ß-actin as a loading control (middle panel). **C** Immunofluorescence for the neuronal marker ßIII tubulin (red) and the neural progenitor marker nestin (green) and quantification of both types of cells at the end of the 6 days differentiation protocol. Cell nuclei were stained with DAPI (blue), scale bar: 10 µm

We previously reported the development and characterisation of a novel neuronal model system to study FENIB based on in vitro transgenesis and differentiation of NPCs obtained from the developing cerebral cortex of E13.5 mouse embryos [17]. These cells can be induced to differentiate into immature neurons by culturing them in a chemically defined medium for six days (see Methods and [17, 26, 29]). We assessed the efficiency of neuronal differentiation of *naïve* (non-transfected) NPCs by looking at the expression of the neuroblast marker *Dcx* by real-time RT-PCR and found a strong upregulation in differentiated cells, reaching comparable transcript levels to those detectable in explants of E13.5 mouse telencephalon, in which cortical neurogenesis is at its peak (Fig. 1A, [30]).

With the scope of limiting the number of passages in in vitro culture and to test the reproducibility of our FENIB model, we recreated the four transgenic lines reported in our previous work [17], with constitutive expression of WT, strongly polymerogenic G392E or delta NS (a truncated version of the protein that misfolds and gets degraded by ERAD but does not polymerise, [13]), as well as the GFP line used as a control for the effects of heterologous protein overexpression. We verified the expression and conformational state of these NS variants by SDS and non-denaturing PAGE and western blot analysis (Fig. 1B) with the same results reported before [17]. In western blot after SDS-PAGE (upper panels), WT NS was detected as a single band of nearly 55 kDa both in the cell lysates and the culture medium, confirming its correct expression and secretion. Instead, G392E NS appeared as a double band in the cell lysate and was absent from the culture medium, confirming a very poor secretion of this mutant variant; the double band is due to differently glycosylated species, with the bottom one having two N-glycans and the top one three, as previously described [16]. Delta NS was detected as a protein of lower molecular weight only in the lysate of cells treated with the proteasome inhibitor MG132, as expected for this truncated protein that in basal conditions is completely degraded by the proteasome, as reported in previous work [13]. By western blot analysis after non-denaturing PAGE (lower panels), WT NS was strongly detected in the cell lysate and weakly in the culture medium as a predominant band corresponding to the monomeric protein plus a fainter lower band; since this sample runs as a single band in SDS-PAGE, the lower band may correspond to a small proportion of monomeric WT NS that has transformed into the latent conformation typical of serpins (full-length protein with the reactive centre loop inserted into ß-sheet A), which confers a compact folding that migrates faster in non-denaturing conditions but runs exactly as the native conformation (the active protease inhibitor) after SDS denaturation [31]. The G392E variant of NS was detected as polymer ladders both in the cell lysate and the culture medium. These observations confirm the reproducibility of our neuronal model and its relevance for FENIB studies.

We next evaluated the efficiency of neuronal differentiation for the four cell lines by immunofluorescence microscopy analysis using the neuronal marker ßIII tubulin. We found comparable results for all the lines, showing that nearly 50% of cells could be identified as neurons positive to this marker and characterised by small nuclei and bipolar neurites. The remaining cells, negative to ßIII tubulin, had a different morphology with bigger nuclei and multiple neurites, reminiscent of glial/astrocyte cells, but were negative to the typical glial cell markers GFAP and S100ß, and were still positive to the NPC marker nestin (Fig. 1C); for this reason, we refer to them as non-neuronal cells. Unless indicated otherwise, the analyses presented in this work have been performed in differentiated cells identified as neurons by ßIII tubulin staining and/or by morphological criteria based on their smaller nuclear size and characteristic bipolar neurite extensions.

### G392E NS neurons show alterations in mitochondrial distribution that are not due to the presence of extracellular G392E NS polymers

**Fig. 2:**
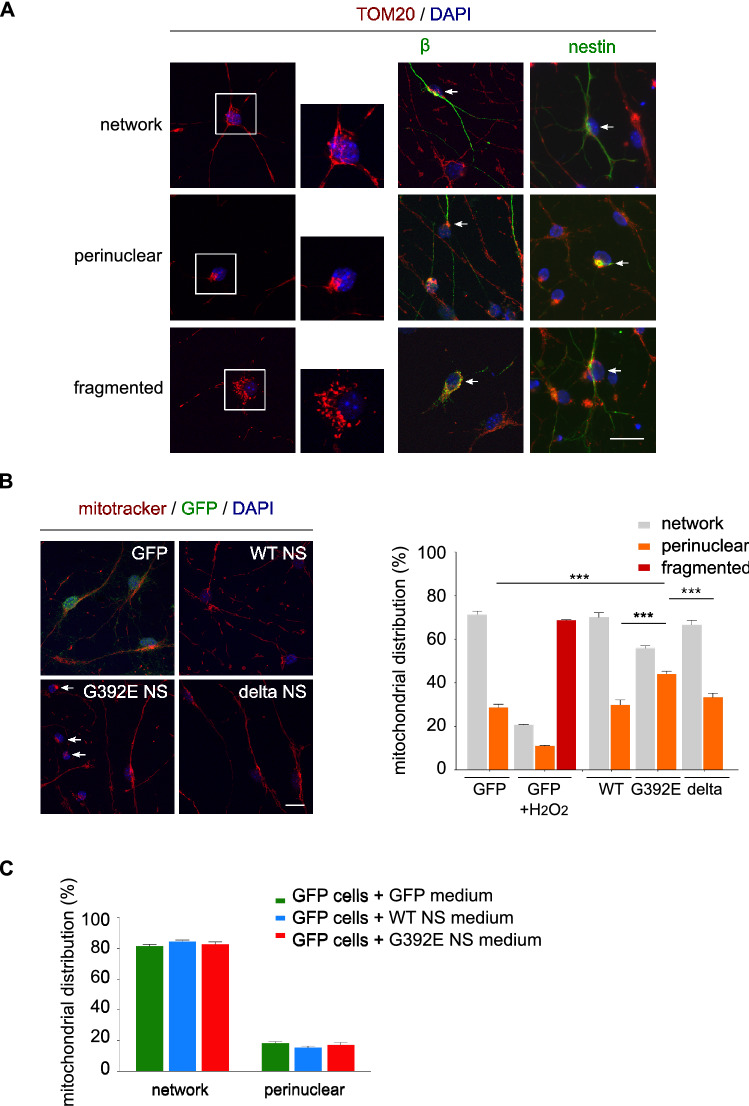
G392E NS neurons show alterations in mitochondrial distribution that are not due to neuroserpin polymers in the culture medium. **A** NPCs overexpressing WT NS were differentiated for 6 days and mitochondrial distribution was analysed by immunostaining and confocal microscopy using an anti-TOM20 antibody (red) alone or in combination with the cell identity markers ßIII tubulin (neuronal marker) and nestin (neural progenitor marker) (green). In the bottom row (fragmented), cells were treated with H_2_O_2_ to induce mitochondrial fragmentation. Three phenotypes were defined: network (top row), with filamentous mitochondria distributed throughout the cells; perinuclear (middle row), with mitochondria clustered close to the nucleus; and fragmented (bottom row), with small round mitochondria located in the perinuclear region. In all panels, cell nuclei were stained with DAPI (blue) and only the merged images are shown (the separate channels are shown in Suppl. Fig. S1). The arrows point to cells positive to the indicated marker and mitochondrial phenotype. Scale bar: 10 µm. **B** NPCs overexpressing GFP or WT, G392E or delta NS were differentiated and stained with Mitotracker to quantify the number of neurons for each mitochondrial phenotype. As a positive control, GFP cells were treated with H_2_O_2_ that mostly induced mitochondrial fragmentation. Cell nuclei were stained with DAPI (blue). The arrows point to cells with perinuclear mitochondria. Scale bar: 10 µm. The data in the graph (collected in Table 1) are mean ± SEM of 3 independent experiments, with 100 to 250 cells of each type counted in each experiment. The percentage of neurons with perinuclear distribution for G392E NS cells was compared to the other cell lines by *t* test: ***p ≤ 0.001. **C** GFP, WT NS and G392E NS NPCs were differentiated for 3 days, after which the culture media were collected and used to treat parallel GFP cultures for the remaining 3 days of differentiation as indicated in the figure. Mitochondrial distribution was assessed by staining with Mitotracker. The data are mean ± SEM of 5 independent experiments, with 100 neurons counted in each repeat

Since we previously observed chronic oxidative stress in neurons expressing polymerogenic G392E NS, we decided to analyse the mitochondrial network in neurons differentiated from the transgenic NPC lines described above, by staining the cells with an antibody against the mitochondrial protein TOM20, an import channel of the outer mitochondrial membrane. Based on the literature and our analysis of the cellular phenotypes, we defined three categories for mitochondrial distribution (Fig. 2A): (i) network, with mitochondria homogeneously distributed throughout the cytoplasm forming a filamentous network within neurons (top row); (ii) perinuclear, with mitochondria that appeared clustered in the neuronal soma and generally positioned on one side of the nucleus (middle row); and (iii) fragmented, when mitochondria appeared small and rounded also located close to the nucleus (bottom row). These three phenotypes were identified for ßIII tubulin-positive neurons (Fig. 2A, ßIII tubulin panels) as well as nestin-positive non-neuronal cells (Fig. 2A, nestin panels). For each transgenic cell line, we quantified the fraction of neurons in each category of mitochondrial distribution by staining the cells with Mitotracker Red CMXRos © (Mitotracker), a red fluorescent dye that labels mitochondria within living cells (Fig. 2B, panels and graph, and Table 1). In basal conditions, WT and delta NS neurons mostly showed the network distribution, indicative of healthy mitochondria. Neurons expressing G392E NS showed a higher proportion of perinuclear mitochondria (above 40%, arrows in Fig. 2B, G392E NS panel) when compared to the other cell lines. Neurons with fragmented mitochondria were not detected in any of the transgenic cell lines but were easily found in control GFP neurons exposed to exogenous H_2_O_2_, indicating that strong oxidative stress causes mitochondrial fragmentation. These results show that the mitochondrial network of these neurons is sensitive to oxidative stress and that G392E NS neurons suffer mitochondrial alterations, probably due to moderate oxidative stress as shown by our previous results [17].

**Table 1:** Mitochondrial distribution in NPCs differentiated for 6 days

Neurons					
	Basal conditions		DEM 50 $\upmu$ M		
	Network	Perinuclear	Network	Perinuclear	Fragmented
GFP	71.32 ± 1.55	28.67 ± 1.56	61.49 ± 0.36	28.56 ± 0.68	10.15 ± 0.56
WT NS	70.03 ± 2.23	29.96 ± 2.23	58.63 ± 2.73	33.09 ± 3.88	8.27 ± 1.18
G392E NS	55.89 ± 1.14	44.10 ± 1.23	49.87 ± 2.45	33.70 ± 2.84	16.42 ± 0.39
dNS	66.69 ± 2.00	33.30 ± 2.00	48.56 ± 3.21	31.80 ± 1.10	12.37 ± 0.45

	Tocopherol 100 $\upmu$ M		Melatonin 100 $\upmu$ M	
	Network	Perinuclear	Network	Perinuclear
GFP	71.94 ± 1.55	28.05 ± 1.55	79.21 ± 1.28	20.78 ± 1.73
WT NS	73.65 ± 2.42	26.34 ± 2.42	74.95 ± 0.68	25.04 ± 1.68
G392E NS	72.50 ± 2.81	27.49 ± 2.81	73.27 ± 1.16	26.72 ± 2.84
dNS	65.26 ± 1.06	34.73 ± 1.07	75.12 ± 2.07	24.87 ± 2.07

Non-neuronal cells					
	Basal conditions		DEM 50 $\upmu$ M		
	Network	Perinuclear	Network	Perinuclear	Fragmented
GFP	86.95 ± 5.22	13.05 ± 5.22	34.86 ± 8.56	16.33 ± 4.48	48.81 ± 4.49
WT NS	89.66 ± 1.70	10.34 ± 1.70	40.10 ± 11.11	20.05 ± 7.47	45.68 ± 11.45
G392E NS	87.20 ± 3.28	12.80 ± 3.28	45.31 ± 13.21	14.90 ± 9.82	39.79 ± 10.21
Dns	90.49 ± 2.16	9.51 ± 2.16	39.91 ± 7.15	17.04 ± 7.31	43.05 ± 0.81

NPCs overexpressing GFP or WT, G392E or delta NS were differentiated for 6 days, treated or not with DEM (50 µM 1 h), melatonin (100 µM 48 h) or tocopherol (100 µM 48 h) and stained with Mitotracker, then counted under the fluorescence microscope to quantify the number of neurons and non-neuronal cells with each type of mitochondrial distribution as described in Fig. 2A. The data are mean ± SEM of 3 independent experiments, with 100–250 cells of each type counted in each experiment

Since polymers of NS have been consistently found in the culture medium of G392E NS cells in this (Fig. 1B, non-denaturing PAGE media panel) and other cellular models of FENIB [7, 17, 32], we investigated their potential effects in mitochondrial distribution. We first quantified the amount of G392E NS polymers in the culture media by sandwich ELISA using a monoclonal antibody with high affinity for polymeric NS (7C6, [7]), and found an average concentration of 1.25 ng/ml. Next, we differentiated GFP NPCs for three days, followed by treatment for another three days with culture medium collected from parallel GFP, WT and G392E NS differentiated cultures. Mitotracker staining of treated GFP cells showed no significant changes in mitochondrial distribution for any of the three conditioned media tested (Fig. 2C), suggesting that the mitochondrial phenotype described above for G392E NS neurons is caused by the intracellular polymerogenic mutant protein.

### G392E NS polymers are similarly present in neurons and non-neuronal cells, but cause mitochondrial alterations mainly in neurons

**Fig. 3:**
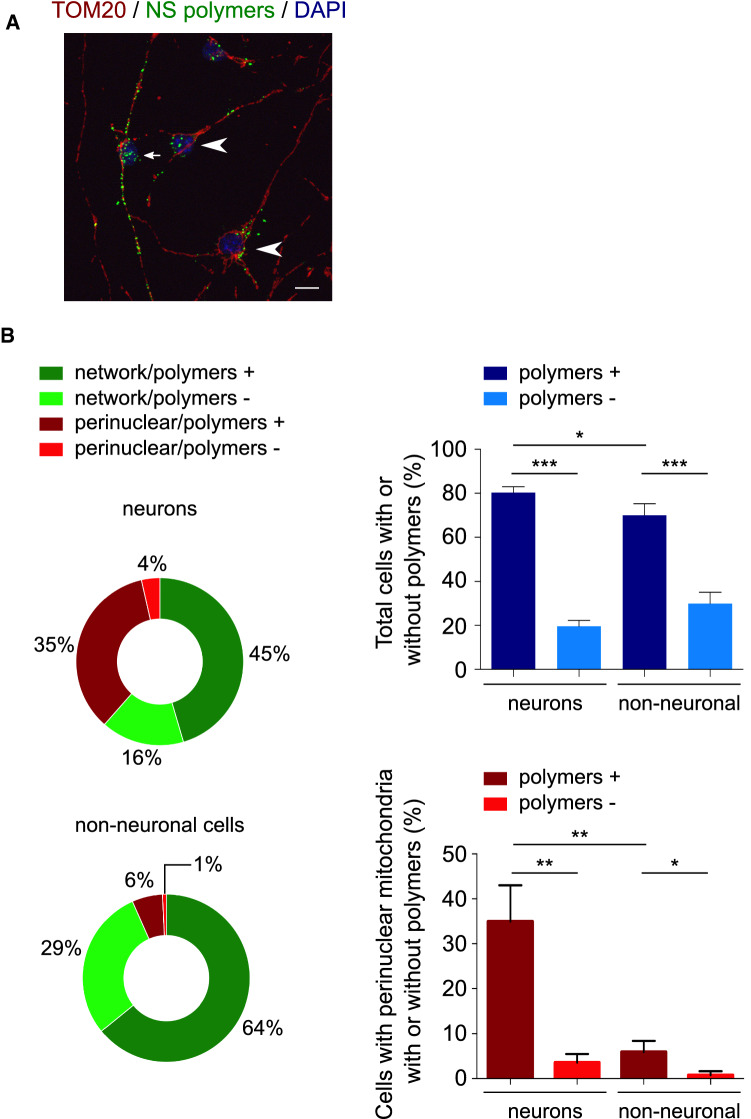
Neurons and non-neuronal G392E NS cells contain NS polymers but alterations in mitochondrial distribution are more frequent in neurons. **A** Immunofluorescence staining of G392E NS cells for NS polymers (anti-polymer mAb 7C6, green) and mitochondria (anti-TOM20, red). The panel shows a neuron (small arrow) and non-neuronal cells (arrowheads) that contain NS polymers. Scale bar: 10 µm. **B** Quantification of neuronal and non-neuronal G392E NS cells with network or perinuclear mitochondrial distribution and with presence or absence of NS polymers. The ring graphs show the percentages of cells for each phenotype (approximated to the nearest whole number for simplicity), the top histogram compares the percentages of neurons and non-neuronal cells with and without NS polymers, and the bottom histogram compares the percentages of neurons and non-neuronal cells with perinuclear mitochondrial distribution that contain or not NS polymers. The data are mean ± SEM of 3 independent experiments, with 100 to 250 cells of each type counted in each experiment; *t* test: ***p ≤ 0.001, **p ≤ 0.01, *p ≤ 0.05. These data are collected in Table 2

We next assessed the mitochondrial distribution of non-neuronal cells in our differentiated cultures and found no significant differences in the number of non-neuronal cells with network and perinuclear distribution when comparing the four cell lines (Table 1 and Suppl. Fig. S2, basal conditions). In order to understand the relationship between mitochondrial distribution, determined by immunofluorescence for TOM20, and the presence of NS polymers, detected by co-staining with the 7C6 monoclonal antibody [7], we analysed both phenotypes in neuronal and non-neuronal G392E NS cells after 6 days of differentiation (Fig. 3A). The percentages of cells presenting each phenotype are reported in Fig. 3B (ring graphs) and Table 2. The fraction of 7C6 positive and negative cells was similar for neurons and non-neuronal cells, with a small increase in polymer-positive neurons over non-neuronal cells (Fig. 3B, top histogram), indicating G392E NS polymer accumulation in both cell types. In contrast, neurons presented a higher proportion of cells with perinuclear mitochondrial distribution (almost 40%, consistent with Fig. 2B) compared to nearly 7% for non-neuronal cells, and this alteration was found specially in the presence of NS polymers (Fig. 3B, bottom histogram), suggesting a higher neuronal sensitivity to the expression of polymerogenic NS.

**Table 2:** Mitochondrial distribution and presence or absence of NS polymers in NPCs differentiated for 6 days

Neurons	% of total cells	Non-neuronal cells	% of total cells
Network 7C6 +	45.43 ± 9.77	Network 7C6 +	64.14 ± 3.80
Perinuclear 7C6 +	34.92 ± 8.10	Perinuclear 7C6 +	5.91 ± 2.45
Network 7C6-	16.08 ± 2.18	Network 7C6-	29.21 ± 4.33
Perinuclear 7C6-	3.56 ± 1.93	Perinuclear 7C6-	0.75 ± 0.91

NPCs overexpressing G392E neuroserpin were differentiated for 6 days and immunostained for NS polymers (anti-polymer mAb 7C6, green) and mitochondria (anti-TOM20, red), then counted under the fluorescence microscope to quantify the percentage of neuronal and non-neuronal cells with or without NS polymers and network or perinuclear mitochondrial distribution. The table presents the percentage of each phenotype for each cell type. The data are mean ± SEM of 3 independent experiments, with 100–250 cells of each type counted in each experiment

### Oxidative conditions enhance and antioxidant molecules rescue the alterations in mitochondrial distribution of G392E NS neurons

**Fig. 4:**
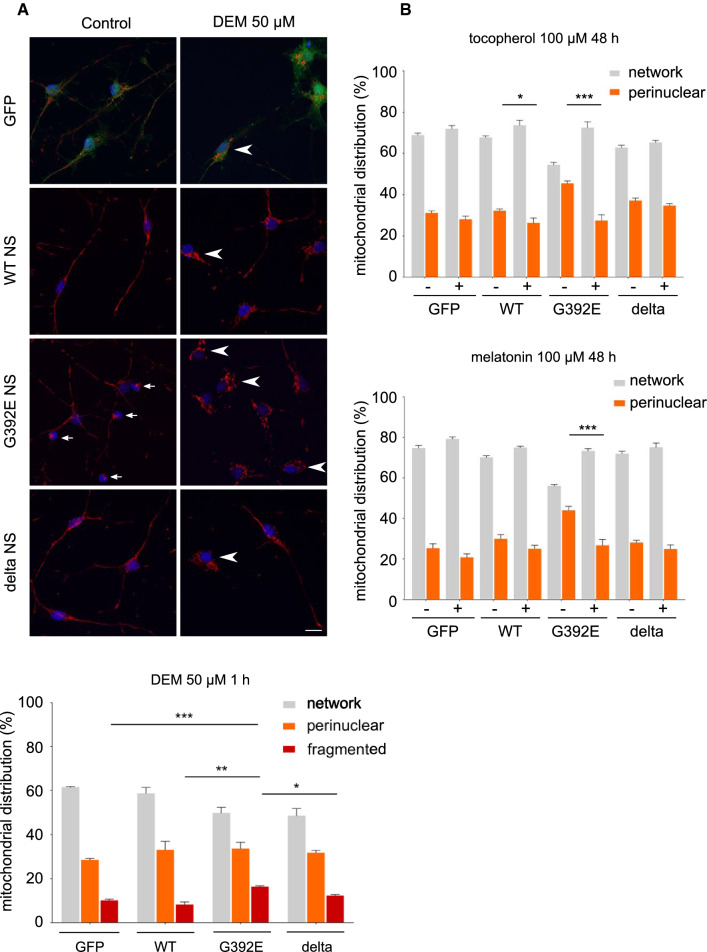
Mitochondrial distribution in differentiated G392E NS neurons can be rescued by antioxidant molecules. **A** The percentage of neurons with network, perinuclear (small arrows) and fragmented (arrowheads) mitochondrial distribution was evaluated for each cell line after differentiation for 6 days, without and with treatment with DEM (50 µM for 1 h), by staining with Mitotracker and cell counting. Cell nuclei were stained with DAPI (blue). Scale bar: 10 µm. The graph shows the mean ± SEM, *n* = 3 (100 cells counted per experiment); *t* test: ***p ≤ 0.001, **p ≤ 0.01, *p ≤ 0.05. **B** Cells prepared as in A, were treated with 10 µM α-tocopherol (top graph) or 10 µM melatonin (bottom graph) during the last 48 h of differentiation and mitochondrial distribution was analysed in the same way. The data are mean ± SEM, *n* = 5 (100 cells counted per experiment); *t* test: ***p ≤ 0.001, *p ≤ 0.05. The data presented in **A** and **B** are collected in Table 1

Our previous results [17] suggest that oxidative stress is related to the altered mitochondrial distribution observed in the present work in G392E NS neurons. To further investigate this, we modulated the levels of oxidative stress in differentiated cultures with pharmacological reagents. We first treated differentiated cells with DEM (diethyl maleate), which depletes cellular glutathione (GSH), an important antioxidant metabolite involved in the mechanism of action of several enzymes upregulated in our G392E NS cells [17]. Neuronal cell counting at the fluorescence microscope of differentiated cultures stained with Mitotracker after DEM treatment revealed the appearance of fragmented mitochondria for all four cell lines, with a significantly higher number of neurons with this phenotype for the G392E NS line (Fig. 4A and Table 1). These results demonstrate that, in our culture conditions, cells can respond to an increase in oxidative stress, and support a higher susceptibility of G392E NS neurons to increased oxidative stress that may be attributed to the presence of NS polymers. When non-neuronal cells were evaluated in the same experiments, no significant differences were found among the four cell lines (Table 1 and Suppl. Fig. S2, DEM 50 µM treatment).

To decrease oxidative stress in differentiated cultures, we used two different antioxidants: melatonin, a hypophysial neurohormone that mediates photoperiodicity in mammals, and α-tocopherol, a vitamin that functions as a lipid-soluble antioxidant protecting cell membranes from oxidative damage. We first evaluated their effects on differentiated WT and G392E NS cells by performing dose–response curves after 24 and 48 h of treatment, followed by staining with Mitotracker and quantification of the fraction of neurons with altered mitochondrial distribution (data not shown). After determining the most effective conditions, we treated all four transgenic lines with 10 µM of each compound during the last 48 h of the 6 days differentiation protocol. As shown in Fig. 4B, neurons expressing GFP, WT and delta NS showed very mild changes in mitochondrial distribution, whereas those expressing G392E NS showed a significant reduction in the percentage of neurons with perinuclear distribution, which decreased to similar values to those observed in control cells. These results support the hypothesis that increased levels of oxidative stress are related to the mitochondrial alterations caused by expression of G392E NS in neurons.

### G392E NS neurons show a reduction of the inner mitochondrial membrane potential but not an increase in mitophagy when compared to WT NS neurons

**Fig. 5:**
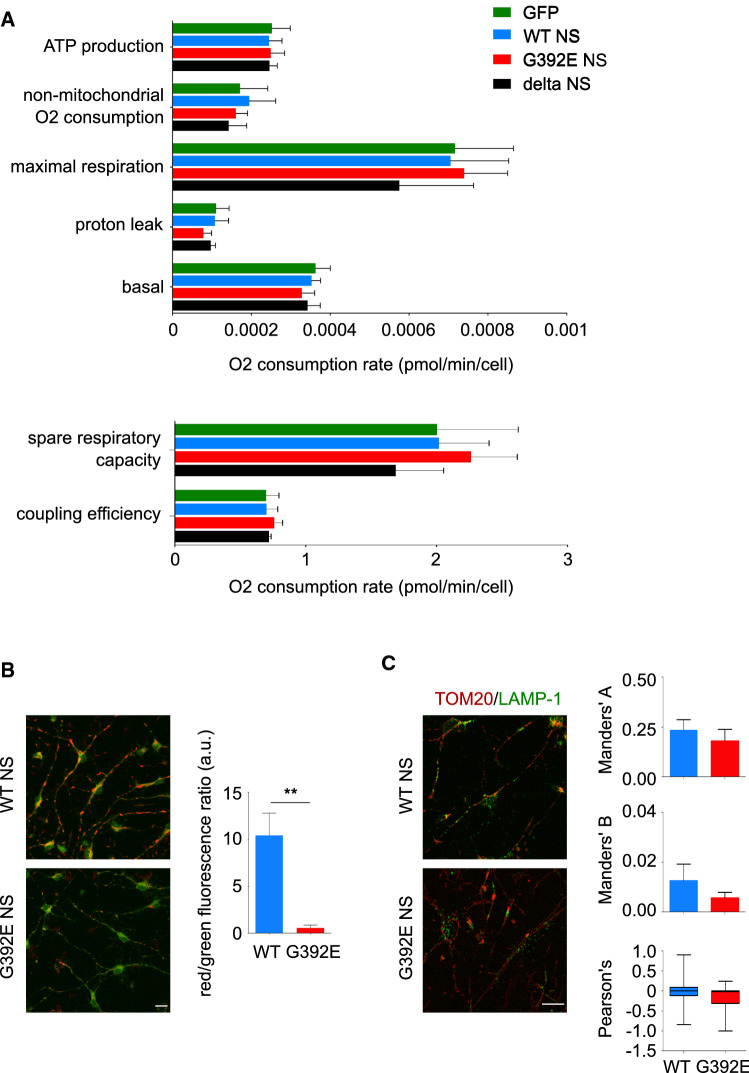
G392E NS neurons present a reduction in the potential of the inner mitochondrial membrane in the absence of alterations of the mitochondrial metabolism. **A** NPCs were differentiated for 6 days and Seahorse analysis was performed by recording the oxygen consumption rate in each culture well, in the presence of pharmacological treatments as described in the Materials and Methods and supplementary Fig. S3. The graphs show the values for mitochondrial metabolic parameters for the four cell lines. The data are mean ± SEM, *n* = 5, one-way ANOVA showed no statistically significant differences between the four cell lines. **B** WT and G392E NS cells were differentiated for 6 days and the inner mitochondrial membrane potential was assayed with the JC-1 probe by live cell imaging and quantified as the ratio of red (aggregated JC-1)/green (monomeric JC-1) fluorescence. Scale bar 10 µm. The data are mean ± SEM, *n* = 3 (at least 100 neurons were analysed in each experiment); *t* test: **p ≤ 0.01. **C** WT and G392E NS cells differentiated for 6 days were stained against the mitochondrial protein TOM20 (red) and the endo-lysosomal protein LAMP-1 (green) and neurons were analysed by confocal microscopy; the co-localisation between both markers was quantified by Manders’ overlap coefficients (**A** green/red; **B** red/green) and Pearson’s correlation coefficient. Scale bar 10 µm. For both Manders’ coefficients, the data are mean ± SEM, for the Pearson’s coefficient data are median with whiskers from maximum to minimum, *n* = 3 (12 fields analysed in each experiment)

To investigate whether the changes in mitochondrial distribution of G392E NS neurons correlated with functional alterations, we assessed the main parameters of mitochondrial respiration in cultures of differentiated GFP, WT NS, G392E NS and delta NS cells. Using a Seahorse platform, oxygen consumption rates (OCR) were measured before and after the addition of pharmacological compounds (described in Material and Methods); these values were used to derive several parameters of mitochondrial respiration, as explained in Suppl. Fig. S3. As reported in Fig. 5A, none of the parameters investigated showed significant variations among the four transgenic cell lines. Since these measures were taken at the level of whole cell populations, with neurons making up nearly 50% of the cells, any changes related to the neuronal mitochondrial phenotype may be masked by the metabolism of non-neuronal cells.

To evaluate mitochondrial function specifically in G392E NS neurons, we decided to investigate the potential of the inner mitochondrial membrane in these cells comparing them to WT NS neurons. We used the cationic fluorescent dye 5,5′,6,6′-tetrachloro-1,1′3,3′-tetraethylbenzamidazol-carboncyanine (JC-1), which is readily transported and accumulates into the matrix of healthy mitochondria forming red fluorescent aggregates but fails to accumulate within depolarised mitochondria, remaining as green fluorescent monomers in the cytosol. Confocal microscopy imaging of living cells and quantification of the red/green ratio showed a higher proportion of green fluorescence in G392E NS neurons compared to the WT NS cells (Fig. 5B), suggesting functional alterations in the mitochondria of neurons expressing polymerogenic G392E NS.

To understand if the mitochondrial alterations observed in G392E neurons were enough to activate mitophagy, we assessed this process by co-staining of differentiated WT and G392E NS cultures with antibodies against the mitochondrial protein TOM20 and the endo-lysosomal protein LAMP-1. Confocal microscopy analysis of WT and G392E NS neurons showed low levels of co-localisation between these two proteins, with similar values for all three co-localisation coefficients for both cell types, indicating that mitophagy is not activated in G392E NS neurons over the levels of WT NS neurons (Fig. 5C).

### G392E NS neurons show a reduction in MERCs and in proteins involved in Ca^2+^ transfer at MERCs

**Fig. 6:**
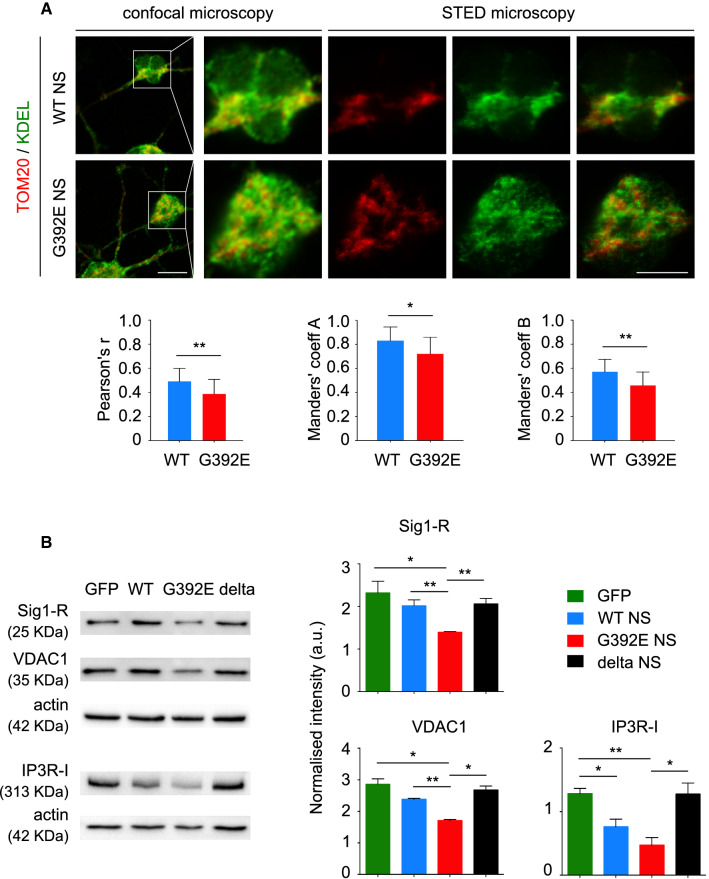
G392E NS neurons show a reduction in mitochondria-associated ER membranes. **A** NPCs overexpressing WT or G392E NS were differentiated for 6 days, double stained for TOM20 (red) and KDEL (green) and analysed by high resolution (STED) confocal microscopy. The panels show representative images of WT and G392E NS cells, while the graphs show the Pearson’s (left) and the two Manders’ coefficients (coefficient A: overlapping of red over green fluorescence, coefficient B: overlapping of green over red fluorescence). Scale bar 10 µm. The data are mean ± SEM, *n* = 3 (15–20 neurons analysed per experiment, for a total of 45–60 cells per condition), *t* test: **p ≤ 0.01, *p ≤ 0.05. **B** WT and G392E NS cell lysates were resolved by 10% w/v acrylamide SDS-PAGE and analysed by western blot with anti-Sigma1-R (Sig1-R), VDAC1 and IP3R-I antibodies. The same membranes were probed for ß-actin as a loading control. The graphs show the densitometric analysis for each protein normalised to ß-actin. The data are mean ± SEM, *n* = 3; *t* test: ***p ≤ 0.001, **p ≤ 0.01, *p ≤ 0.05

Since NS is synthesised in the ER and aberrant polymers accumulate in its lumen, we also investigated the crosstalk between ER and mitochondria by assessing the extent of their contact regions, comparing the MERCs between G392E NS and WT NS neurons. We performed double immunofluorescent staining for TOM20 and KDEL (the ER retention motif found in many ER resident proteins) in differentiated neurons and used high-resolution STED (stimulated emission depletion) confocal microscopy to quantify MERCs (Fig. 6A). The three coefficients used in our analysis, presented in Fig. 6A, showed a small but significant reduction in the extent of contact regions between the ER and mitochondria in G392E NS neurons when compared to the WT NS ones, suggesting that neuronal expression of polymerogenic NS alters the crosstalk between the ER and mitochondria.

We sought confirmation of this observation by quantifying the levels of three membrane proteins enriched at MERCs and involved in Ca^2+^ transport between the two organelles: two ER membrane proteins, sigma-1 receptor (Sig1-R) and inositol 1,4,5-triphosphate receptor (IP3R-I), and one protein of the outer mitochondrial membrane, voltage-dependent anion channel (VDAC1) [33]. As shown in Fig. 6B (panels and densitometry graphs), SDS-PAGE and western blot analysis of cell lysates from WT and G392E NS differentiated cultures showed significantly lower amounts of the three proteins in the G392E NS lysates when compared to GFP, WT NS and delta NS cells, supporting our microscopy data and suggesting a functional alteration of MERCs in G392E NS cells.

### G392E NS polymers activate NFκB but not the UPR in differentiated cultures

**Fig. 7:**
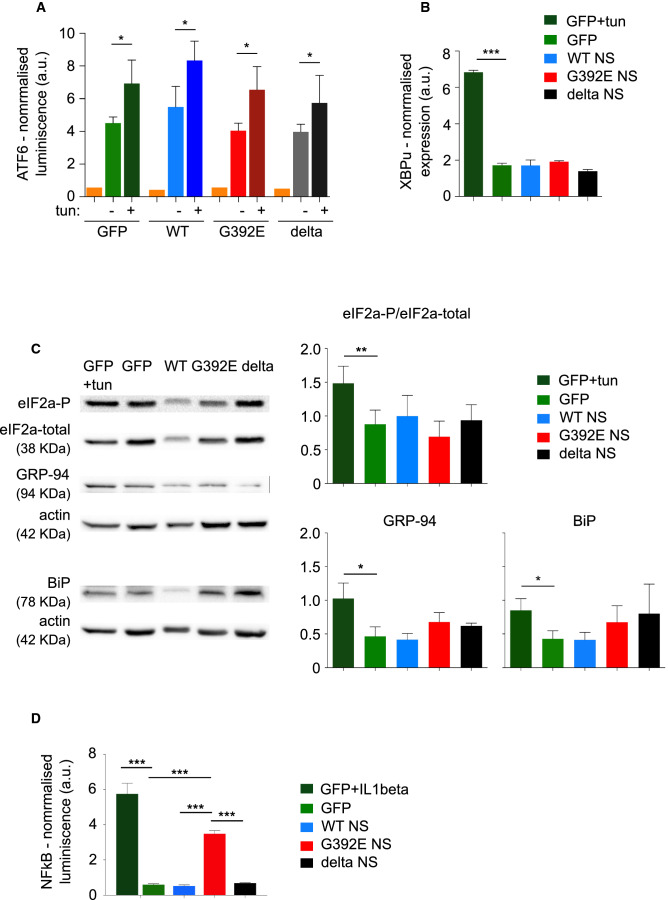
Differentiated cultures of G392E NS cells show activation of NFκB, but not the UPR. **A** NPCs overexpressing GFP or WT, G392E or delta NS were co-transfected with a firefly luciferase reporter responsive to the activation of the UPR and a constitutive Renilla luciferase reporter as a transfection control used for normalisation. Cells were left to recover for 1 day and differentiated to neurons for 3 days. For each line, parallel wells were treated or not with tunicamycin (TUN) for 6 h as a positive control for UPR activation. Cells were collected for a dual luciferase assay and parallel wells without luciferase transfection were used as a baseline control (orange bars). **B** Relative mRNA levels of unspliced XBP1 in cultures expressing GFP or WT, G392E or delta NS after 6 days of differentiation and normalization to expression levels in proliferating GFP cultures. GFP cells treated with tunicamycin for 6 h (GFP + tun) were used as a positive control. **C** NPCs overexpressing GFP or WT, G392E or delta NS were differentiated to neurons for 6 days and collected for western blot analysis after 10% w/v acrylamide SDS-PAGE, using anti-KDEL for BiP and GRP-94 detection, anti-eIF2α and anti-phosphorylated eIF2a antibodies. The first three panels from the top correspond to the ß-actin loading control underneath them (all performed in the same membrane), while the BiP panel refers to its own ß-actin loading control (both in the same membrane). The graphs show the densitometric quantification for each protein normalised to ß-actin. **D** Cells were co-transfected and treated as in **A** except for the use a firefly luciferase reporter for NFκB activation and the use of IL1-ß to generate a positive control for the GFP cell line. All quantitative data are mean ± SEM, *n* = 3; *t* test: ***p ≤ 0.001, **p ≤ 0.01, *p ≤ 0.05

Another important aspect of the interaction between ER and mitochondria is whether ER stress signalling pathways are activated by polymer accumulation. It has been previously reported that NS polymers do not activate the UPR [13, 14] but these studies were performed using non-neuronal cells, so we decided to investigate this in our neuronal model of FENIB. The misfolded variant delta NS, which does not form polymers and is efficiently degraded by the proteasome, has been shown to activate the different branches of the UPR when ERAD is blocked with MG132 to increase the accumulation of delta NS in the ER [13]. Our attempts to use delta NS as a positive control in the assays described below were unsuccessful due to the high toxicity of MG132 in this cellular system. We first quantified the activation levels of a luciferase reporter expressed under the control of ER stress elements (ERSE); as shown in Fig. 7A, there was no difference in luciferase levels among the four transgenic cell lines, although they were all able to respond at similar levels to the ER stressor tunicamycin. We next sought confirmation of these results by looking at specific UPR branches and downstream effectors. We evaluated the IRE1 (inositol-requiring enzyme 1 α) branch by quantifying the transcript level of the unspliced and spliced versions of XBP1 (X-box binding protein 1) by real-time RT-PCR; we found no amplification of the spliced form of XBP1 (data not shown), but we observed a strong upregulation of unspliced XBP1 expression in control GFP cells treated with tunicamycin (Fig. 7B). We assessed the PERK (protein kinase R-like ER kinase) branch by looking at the ratio of phosphorylated eIF2α (eukaryotic initiation factor 2 α) by western blot, using antibodies against the phosphorylated and total versions of the protein; again, we found no differences among the four cell lines, although tunicamycin was able to increase eIF2α phosphorylation in GFP control cultures in our experimental conditions (Fig. 7C, eIF2α panels and graph). Finally, we analysed the protein levels of two ER-resident chaperones, GRP94 (glucose-regulated protein 94) and BiP (binding immunoglobulin protein)/GRP78, by western blot with an anti-KDEL antibody, and found no significant differences for either protein (Fig. 7C, GRP94 and BiP panels and graphs).

Using PC12 (pheochromocytoma) cells, we previously reported that NS polymers activate the transcription factor NFκB (nuclear factor κ-light-chain-enhancer of activated B cells) as part of an independent ER stress response pathway known as EOR (ER overload response [13]). We assessed this in differentiated cultures by using a luciferase reporter for NFκB activation, which was co-transfected with a control reporter for transfection efficiency. We found a strong activation in G392E NS cells compared to GFP, WT and delta NS cells, albeit at lower levels than those achieved in GFP control cells treated with interleukin 1ß (1ß IL) used as a positive control (Fig. 7D). Taken together, these results confirm in neuronal cells previous evidence of the activation of EOR, but not UPR, by polymerogenic NS.

## Discussion

FENIB is a rare type of neurodegeneration caused by mutations in NS that lead to its intracellular polymerisation and accumulation within the ER. Although the molecular bases of the disease have been known for some time [2], the cellular consequences of polymerogenic NS expression by neurons are still poorly characterised. Moreover, since both polymer accumulation and the concomitant decrease in active NS secretion take place within the nervous system, it is unclear which of the symptoms that characterise this disease are due to a toxic gain-of-function by ER polymers, and which to a lack-of-function mechanism. A decrease in anti-protease protection by NS has been suggested as the cause for the epileptic component of FENIB under the name of neuroserpinosis [34]. The toxicity of NS polymers has been addressed by expression of polymerogenic variants of human NS in cellular and animal models. Although the studies using transformed mammalian cell lines have not shown clear signs of toxicity, the accumulation of NS polymers in the fly brain reduced their climbing ability [7], and transgenic mice overexpressing polymerogenic mutant NS showed neurological symptoms including ataxia, seizures and behaviour abnormalities [10, 11]. These results support the toxic nature of NS polymer accumulation in neurons, but the mechanisms underlying it are not clear yet.

In recent years, we have developed an in vitro neuronal NS expression system by means of transgenic mouse NPCs with stable integration of a bicistronic cassette driving expression of a puromycin resistance gene and the NS gene in three different versions: WT, the strongly polymerogenic G392E and the truncated delta NS. This allows for continuous expression of NS in puromycin-selected cells, which can be differentiated to neurons with a chemically defined medium applied for six days. With this system, we have previously shown that G392E NS expression leads to the activation of an antioxidant adaptive response, consisting in the upregulation of several antioxidant genes (Aldh1b1, Apoe, Gpx1, Gstm1, Prdx6, Scara3, Sod2), suggesting that G392E NS cells react to an oxidative insult caused by NS polymers; when challenged with drugs that block the antioxidant protective pathways, by glutathione chelation with DEM and catalase inhibition with 3-amino-1,2,4 triazole, G392E NS neurons die by apoptosis at a higher proportion than control neurons, supporting an increased sensitivity to oxidative stress upon conditions that impair the antioxidant response [17]. Here we build on previous studies and show that the oxidative insult correlates with alterations in the mitochondrial network. In physiological conditions, neurons present a well-distributed mitochondrial pattern that extends into all neurites. The expression of G392E NS caused an increase in the percentage of neurons with mitochondrial localisation to the perinuclear area, indicative of alterations in mitochondrial dynamics that can have multiple origins, as seen in other neurodegenerative conditions [35]. We have previously described the presence of G392E NS polymers in the culture medium in this and other cellular systems [7, 12, 13, 17, 36] and have shown that NS polymers are secreted and not released by cell death [32], similarly to polymers of the Z variant of A1AT [37]. Our present results support the idea that mitochondrial alterations are caused by intracellular G392E NS, since the culture medium conditioned by G392E NS cells did not cause the same phenotype in control GFP or WT NS neurons. Furthermore, the perinuclear localisation of mitochondria was worsened to fragmentation by increasing oxidative stress through pharmacological means (DEM), and was ameliorated by antioxidant molecules (tocopherol and melatonin), supporting a link between oxidative stress and mitochondrial alterations caused by NS polymer accumulation in FENIB. This is reminiscent of A1AT deficiency, the most common serpinopathy, for which previous studies have shown an increase in oxidative stress-regulating genes and reactive oxygen species in the liver of transgenic PiZ mice that express the polymerogenic Z variant of A1AT [38], as well as mitochondrial damage in the livers of PiZ mice and human patients and in cell culture models of the disease [39, 40]. Very recently, these observations have been extended to show mitochondrial dysfunction including elevated reactive oxygen species and compromised mitochondrial membrane integrity in PiZ mice [41]. Teckman and collaborators also showed an increase in mitochondrial autophagy in mouse and human hepatocytes [39] that we have not observed in our FENIB model. This may be due to different levels of polymer accumulation, differences in polymer toxicity between A1AT and NS or different cellular responses in liver and neuronal cells. In fact, previous studies support the notion that neurons, being postmitotic cells, do not activate mitophagy as easily as other cell types to avoid cell death [42]. The presence of mitochondrial alterations is a common feature of many neurodegenerative conditions, as seen for Parkinson’s and Alzheimer’s disease, amyotrophic lateral sclerosis and others, to the extent that mitochondrial perturbation has been suggested as a main component and relevant drug target in neurodegeneration [43]. Different types of mitochondrial alterations have been described depending on the pathology, the animal or cell culture model used and the stage of the disease, with a heterogenicity that requires further research to complete our understanding.

Our results also show a different sensitivity to NS polymers in neurons and non-neuronal cells; although both cell types contained polymers as detected by immunofluorescence, the percentage of cells with altered mitochondrial distribution was much higher in neurons. This is consistent with the higher resistance of non-neuronal cells to oxidative insults [44]. Within the nervous system in vivo, NS is expressed in several types of neurons but not in non-neuronal cells, suggesting that our results are relevant to FENIB. Also, the alterations in mitochondrial distribution observed in G392E NS neurons did not correlate with a clear functional disruption of mitochondrial respiration in our cell culture model as measured by Seahorse assay. This may be partially due to the fact that this assay analyses the whole population of cultured cells in each tissue culture well, including the non-neuronal cells that account for approximately half of the population. By looking specifically into G392E NS neurons by confocal microscopy, we could detect a decrease in the inner mitochondrial membrane potential that was compatible with cellular viability, since our cultures did not show evidence of cell death. Our results suggest that the mitochondrial perturbations observed in our cell culture model, although indicative of cellular suffering, are a sign of mild toxicity compatible with normal levels of mitochondrial metabolism and neuronal survival.

One important aspect of NS polymer toxicity is how the abnormal accumulation of a protein within the ER is communicated to the rest of the cell. Previous studies have shown that the accumulation of A1AT polymers in the ER of cultured cells decreases the mobility of ER proteins without activating the UPR but causing a higher sensitivity to additional stressing events like preventing normal protein glycosylation or glucose deprivation [45]. Other cell culture studies have found a similar lack of UPR activation for A1AT polymers [24, 46], although several components of the UPR signalling pathway were upregulated in peripheral blood monocytes of A1AT deficiency patients [47], suggesting that different cell types may react diversely to the presence of polymers. In the case of FENIB, two studies based on the analysis of transgenic S49P NS mice have reported a limited and transient activation of the UPR [48, 49], in contrast with studies in PC12 and HeLa cells showing a lack of UPR signalling upon overexpression of S52R and G392E NS [13, 14]. These differences may be due to the nature of the expression system, with animal models providing the possibility of looking into the long-term effects of polymers. In the present work, we have reassessed UPR activation reasoning that neurons obtained by in vitro differentiation of non-immortalized NPCs are closer to the in vivo situation and may show a higher susceptibility to NS polymers than transformed cells. Our analysis of several signalling mediators that take part in the UPR has confirmed a lack of activation in differentiated cultures, confirming that NS polymers, like A1AT polymers, are not strong UPR activators. Our present results also confirm that the expression of polymerogenic NS activates NFκB, as seen previously for A1AT [24, 46] and for NS [13]. In the later work, NFκB activation required the presence of cytosolic Ca^2+^, suggesting that NS polymer accumulation within the ER caused a Ca^2+^ leakage from ER stores, but the mechanism behind this was not clarified. Here we have assessed the extent of MERCs, the membrane structures that mediate the communication between the ER and mitochondria, including Ca^2+^ transfer between these two organelles [50]. We found that MERCs, measured as the overlap of ER and mitochondrial staining by high resolution confocal microscopy, were reduced in G392E NS neurons, as well as a reduction in protein levels of several mediators of Ca^2+^ transfer from the ER to mitochondria, namely IP3R-I, VDAC1 and Sig1-R [33]. Alterations in the extent, thickness and function of MERCs have been described in other neurodegenerative diseases including Alzheimer’s and Parkinson’s disease, highlighting the importance of organelle communication in pathological as well as in physiological states [33]. An interesting link between ER-mitochondria communication at MERCs and adaptation to chronic stress in neurons has been recently described, in which a signalling pathway involving Mul1 (or MULAN, mitochondrial ubiquitin ligase activator of NFκB) and Mfn1 (mitofusin 1) maintains mitochondrial integrity and restrains mitophagy in neurons during early stress by regulating mitochondrial morphology and ER-mitochondria contacts [42]. This seems highly relevant in the context of previous and present observations for FENIB, where adaptation to oxidative stress, alterations in mitochondrial distribution and MERCs, and NFκB activation have all been described ([13, 17] and present results).

**Fig. 8:**
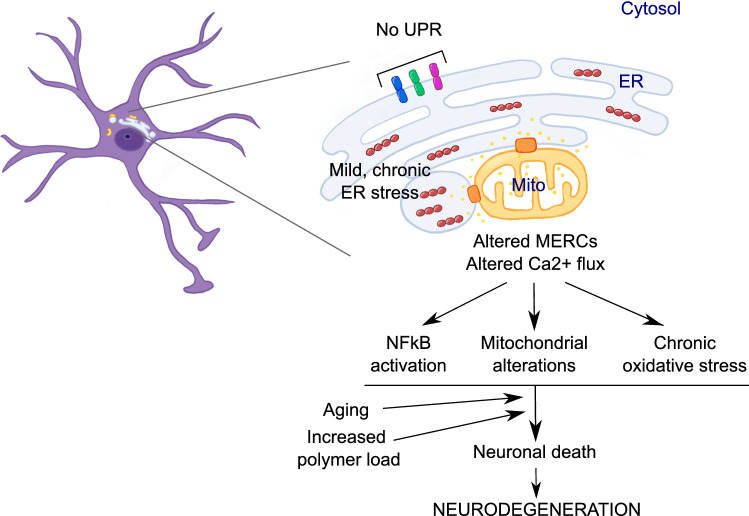
Proposed mechanism for NS polymer toxicity and neurodegeneration in FENIB. The accumulation of NS polymers (red bead chains) within the ER of neurons causes chronic ER stress that is transmitted to mitochondria (Mito) through MERCs (orange transmembrane complexes) in a Ca^2+^-dependent manner (Ca^2+^ ions: small yellow dots). This causes mitochondrial alterations, chronic oxidative stress (which may start at the ER and/or at mitochondria, [51]) and NFκB activation in the absence of UPR signalling (blue, green and fuchsia transmembrane proteins: UPR transducers). Aging and increasing polymer load eventually overcome the neuroprotective responses, causing neuronal death and neurodegeneration

Our results suggest the hypothesis (summarised in Fig. 8) that neurodegeneration in FENIB is due, at least in part, to chronic, mild ER stress caused by the accumulation of NS polymers that alters the communication between ER and mitochondria mediated by MERCs, especially those involved in Ca^2+^ transfer. This in turn affects mitochondria without causing major dysfunction, at least during the initial stage of the disease, when patients present no clinical symptoms. A delay in the onset of clinical manifestations is common to many neurodegenerative diseases, even in the presence of causative mutations [43]. In later stages of FENIB, a decrease of protective cellular mechanisms and/or the continuous deposition of NS polymers would overcome neuronal adaptation to the insult, causing cell death, the onset of clinical disease and finally the death of the patient. Future studies are needed to complete our knowledge of the cellular effects of polymerogenic NS during neuronal development and in mature neurons, as well as during the initial and later stages of neurodegeneration in FENIB.

### Supplementary Information

Below is the link to the electronic supplementary material.

Supplementary Figure S1. Single staining panels for the ßIII tubulin and nestin panels shown in Fig. 2A as merged confocal microscope images. The cellular markers ßIII tubulin and nestin were acquired in the green channel and the TOM20 mitochondrial marker in the red channel. The arrows point to the relevant mitochondrial phenotype indicated for each panel. Scale bar: 10 µm. (EPS 4961 KB)

Supplementary Figure S2. Mitochondrial distribution in non-neuronal cells. NPCs overexpressing GFP or WT, G392E or delta NS were differentiated for 6 days, treated with DEM (50 µM for 1 h) or left untreated (basal conditions) and stained with Mitotracker to quantify the number of non-neuronal cells for each mitochondrial phenotype. The data are mean ± SEM of 5 independent experiments, with 100 neurons counted in each repeat (EPS 179 KB)

Supplementary Figure S3. Seahorse analysis of mitochondrial metabolism in differentiated NPC cultures. NPCs overexpressing GFP or WT, G392E or delta NS were differentiated for 6 days and their oxygen consumption rates were measured under different conditions created by addition of pharmacological reagents (indicated in the graph) following the protocol described in Materials and Methods. Initially, baseline cellular OCR was measured, from which basal respiration was derived by subtracting non-mitochondrial respiration (see below). Next, oligomycin, a complex V inhibitor, was added and the resulting OCR was used to derive ATP-linked respiration (by subtracting the oligomycin OCR from baseline OCR) and proton leak respiration (by subtracting non-mitochondrial respiration from oligomycin OCR). Next, carbonyl cyanide-p-trifluoromethox-yphenyl-hydrazol (FCCP), a protonophore, was added to collapse the inner membrane gradient, allowing the electron transport chain to function at its maximal rate and maximal respiratory capacity was derived by subtracting non-mitochondrial respiration from the FCCP rate. Lastly, antimycin A, an inhibitor of complex III, was added to shut down the electron transport chain function, revealing the non-mitochondrial respiration. The mitochondrial reserve capacity was calculated by subtracting basal respiration from maximal respiratory capacity. The oxygen consumption rate values thus generated were used to calculate the metabolic parameters presented in figure 5A. (EPS 301 KB)

## Data Availability

All the data generated in this study is reported in the figures, tables and supplementary figures that are part of this article.

## Abbreviations

A1AT: α-1 Antitrypsin
bFGF: Basic fibroblast growth factor
BiP: Binding immunoglobulin protein
BSA: Bovine serum albumin
DABCO: 1,4-Diazabicyclo [2.2.2] octane
DAPI: 6-Diamidino-2 phenylindole
DEM: Diethyl maleate
eIF2α: Translation initiation factor 2α
ER: Endoplasmic reticulum
EOR: ER overload response
FCCP: Carbonyl cyanide-*p*-trifluoro methoxyphenyl hydrazine
FENIB: Familial encephalopathy with neuroserpin inclusion bodies
GFP: Green fluorescent protein
GRP94: Glucose-regulated protein 94
hBDNF: Human brain derived neurotrophic factor
hEGF: Human epidermal growth factor
HRP: Horseradish peroxidase
IRE1α: Inositol-requiring enzyme 1α
LAR: Luciferase assay reagent
NS: Neuroserpin
MERCs: Mitochondria-ER contact sites
NFκB: Nuclear factor κB
NPCs: Neural progenitor cells
OCR: Oxygen consumption rate
PAGE: Polyacrylamide gel electrophoresis
PERK: Protein kinase R-like ER kinase
PFA: Paraformaldehyde
SDS: Sodium dodecyl sulphate
TMB: 3,3′,5,5′-Tetrametilbenzidin
UPR: Unfolded protein response
WT: Wild type
XBP1: X-box binding protein 1
